# Efficacy and Renal Safety of Prophylactic Tenofovir Alafenamide for HBV-Infected Cancer Patients Undergoing Chemotherapy

**DOI:** 10.3390/ijms231911335

**Published:** 2022-09-26

**Authors:** I-Cheng Lee, Keng-Hsin Lan, Chien-Wei Su, Chung-Pin Li, Yee Chao, Han-Chieh Lin, Ming-Chih Hou, Yi-Hsiang Huang

**Affiliations:** 1 Division of Gastroenterology and Hepatology, Department of Medicine, Taipei Veterans General Hospital, Taipei 112, Taiwan; 2 School of Medicine, National Yang Ming Chiao Tung University, Taipei 112, Taiwan; 3 Institute of Pharmacology, College of Medicine, National Yang Ming Chiao Tung University, Taipei 112, Taiwan; 4 Division of Clinical Skills Training, Department of Medical Education, Taipei Veterans General Hospital, Taipei 112, Taiwan; 5 Cancer Center, Taipei Veterans General Hospital, Taipei 112, Taiwan; 6 Institute of Clinical Medicine, National Yang Ming Chiao Tung University, Taipei 112, Taiwan

**Keywords:** hepatitis B virus, cancer, chemotherapy, entecavir, tenofovir disoproxil fumarate, tenofovir alafenamide

## Abstract

There are no data comparing the efficacy and safety of prophylactic entecavir (ETV), tenofovir disoproxil fumarate (TDF) and tenofovir alafenamide (TAF) for HBV-infected cancer patients undergoing chemotherapy. This study aimed to compare the efficacy and renal safety of ETV, TDF and TAF in this setting. HBsAg-positive cancer patients treated with ETV (n = 582), TDF (n = 200) and TAF (n = 188) during chemotherapy were retrospectively enrolled. Antiviral efficacy and risk of renal events were evaluated. The rate of complete viral suppression at 1 year was 94.7%, 94.7% and 96.1% in ETV, TDF and TAF groups, respectively (*p* = 0.877). A significant proportion of patients developed renal dysfunction during chemotherapy. The incidences of acute kidney injury (AKI) and chronic kidney disease stage migration were comparable among the ETV, TDF and TAF groups. TAF was relatively safe in patients with predisposing factors of AKI, including hypoalbuminemia and cisplatin use. In patients who were switched from TDF to TAF during chemotherapy, the renal function remained stable and viral suppression was well maintained after switching. In conclusion, TAF had good renal safety and comparable efficacy with ETV and TDF for HBV-infected cancer patients receiving chemotherapy. Switching from TDF to TAF during chemotherapy is safe, without a loss of efficacy.

## 1. Introduction

Hepatitis B virus (HBV) infection remains a prevalent health problem, affecting more than 250 million people around the world [1]. Host immune responses play critical roles in controlling HBV. HBV reactivation is a well-recognized complication in HBV-infected cancer patients undergoing systemic chemotherapy or immunosuppressive therapy, which may result in potentially fatal hepatic decompensation [2,3,4,5]. Current guidelines suggest that HBV-infected cancer patients undergoing chemotherapy should receive prophylactic nucleos(t)ide analogues (NUCs) with high genetic barriers, including entecavir (ETV), tenofovir disoproxil fumarate (TDF) or tenofovir alafenamide (TAF) [6,7,8]. However, there are some challenges to the use of ETV and TDF. While ETV is associated with a high risk of resistance in lamivudine-experienced patients, bone and renal safety issues are a major concern with TDF [9,10]. TAF, a new prodrug of tenofovir, has been shown to have non-inferiority of viral suppression, a higher rate of ALT normalization and significantly better bone and renal safety as compared to TDF [11,12]. In addition, TAF can be substituted for TDF in chronic hepatitis B (CHB) patients for improved safety without a loss of efficacy [13].

Renal dysfunction is an important issue for the management of HBV infection. Cancer patients undergoing chemotherapy are especially vulnerable to renal dysfunction, depending on the type and stage of cancer, the chemotherapy regimen and comorbidities [14,15]. Renal dysfunction in cancer patients is associated with increased morbidity and mortality and increases the risk of adverse events from chemotherapy [14]. The improved renal safety and no need for dosage adjustment in patients with renal function fluctuations might be advantages of TAF for patients undergoing chemotherapy. Nevertheless, currently there is a lack of evidence for the use of TAF in patients undergoing chemotherapy. Moreover, there are no data comparing the efficacy and safety of ETV, TDF and TAF antiviral prophylaxis in this setting. The aim of this study was to compare the efficacy and risk of renal dysfunction during ETV, TDF and TAF antiviral prophylaxis in HBsAg-positive cancer patients undergoing chemotherapy.

## 2. Results

### 2.1. Patient Characteristics

**Figure 1:**
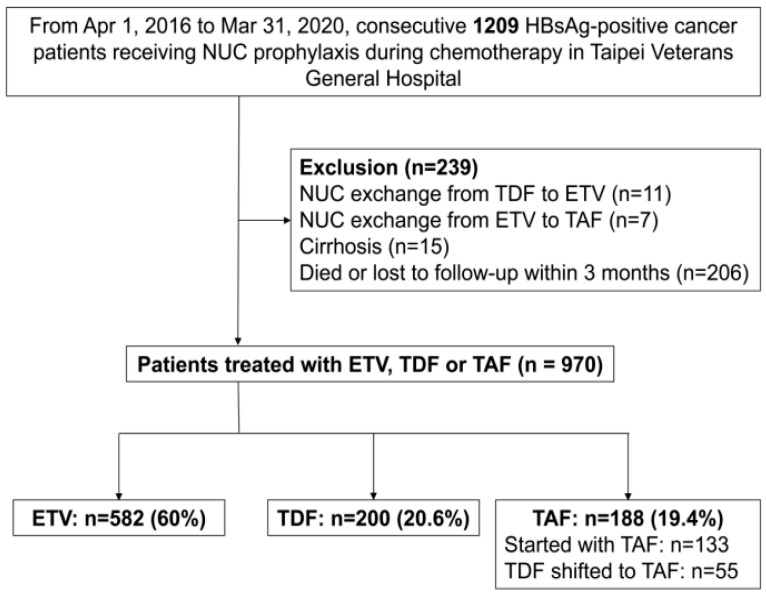
Screening, enrollment and grouping of patients.

A total of 970 patients were enrolled in this study, including 582 patients in the ETV group, 200 in the TDF group and 188 patients in the TAF group (Figure 1). Fifty-five patients in the TAF group were initially treated with TDF and were switched to TAF later. Table 1 shows the baseline characteristics of the three groups of patients. The majority of patients had HBeAg-negative carrier status and were in chronic kidney disease (CKD) stage 1 or 2. Around 61% of patients had an HBV DNA level less than 2000 IU/mL before chemotherapy, and 24% of patients had undetectable HBV viral loads. Gastrointestinal (GI) cancers were the most common cancer types (24.5%), followed by hematological, lung, head and neck and breast cancers. The median follow-up period was 23, 25.4 and 11.9 months in the ETV, TDF and TAF groups, respectively (*p* < 0.001). The ETV group had a significantly higher proportion of hematological cancers, longer chemotherapy and NUC prophylaxis duration, higher baseline blood urea nitrogen (BUN) and creatinine levels, a lower estimated glomerular filtration rate (eGFR) and more patients with an advanced CKD stage, compared to the other two groups of patients.

### 2.2. Antiviral Efficacy and Incidence of Renal Events at One-Year Follow-Up

The antiviral efficacy and incidence of renal events at 1 year after starting NUC therapy in 686 patients with a follow-up of more than 1 year are shown in Table 2. The baseline characteristics of patients with a follow-up of more than 1 year are shown in Table S1. The virological response rate was 94.7%, 94.7% and 96.1% in the ETV, TDF and TAF groups, respectively (*p* = 0.877). Two patients (0.5%) in the ETV group, one (0.7%) in the TDF group and none in the TAF group developed HBV reactivation (*p* = 0.694). Non-compliance with NUC interruptions or premature cessations were noted in these cases. One patient in the ETV group developed HBV-related hepatic decompensation.

The overall incidence of acute kidney injury (AKI) was 13.3% at 1-year follow-up, which was not significantly different among the three NUC groups (*p* = 0.420). There was no significant difference in the incidence of eGFR decrease >30%, CKD stage migration and hypophosphatemia among the three groups. The overall incidence of eGFR below 50 mL/min was 19% at one year, which was significantly higher in the ETV group (24.2%) as compared to the TDF (10.1%) and TAF (11.7%) groups (*p* < 0.001).

In the subgroup patients with CKD stage 1, there was no significant difference in the incidence of AKI, eGFR below 50 mL/min, CKD stage worsening and hypophosphatemia among the three groups, whereas the incidence of eGFR decrease >30% was lower in the TAF group (12.9%) as compared to the ETV (27.2%) and the TDF (29.4%) groups (*p* = 0.044). In patients with CKD stage 2, the incidence of renal events was generally comparable among the three groups, except that the incidence of eGFR below 50 mL/min was higher in the ETV group (28.7%) than the TDF (13.3%) and TAF (16.3%) groups (*p* = 0.027). In patients with CKD stage 3–5, there were no significant differences in the incidence of renal events among the three groups.

### 2.3. Survival Analysis for the Cumulative Incidence of Acute Kidney Injury (AKI)

**Figure 2:**
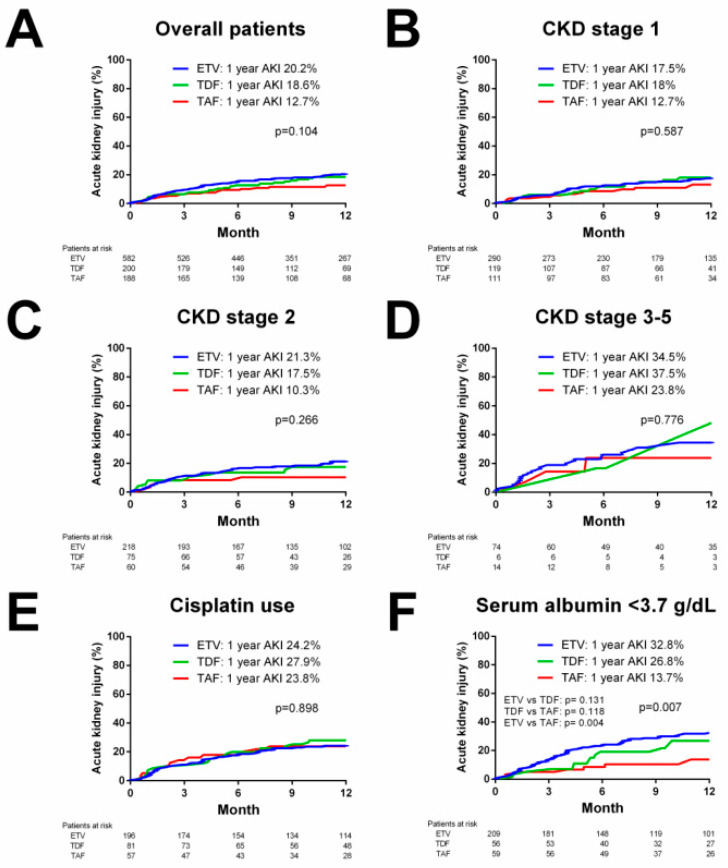
Cumulative incidence of acute kidney injury (AKI) during entecavir (ETV), tenofovir disoproxil fumarate (TDF) and tenofovir alafenamide (TAF) therapy. (**A**) Cumulative incidence of AKI in overall patients. (**B**) Cumulative incidence of AKI in subgroup patients with CKD stage 1. (**C**) Cumulative incidence of AKI in subgroup patients with CKD stage 2. (**D**) Cumulative incidence of AKI in subgroup patients with CKD stage 3–5. (**E**) Cumulative incidence of AKI in subgroup patients with cisplatin use. (**F**) Cumulative incidence of AKI in subgroup patients with serum albumin <3.7 g/dL.

The whole cohort of 970 patients was included for the survival analysis of AKI. By Kaplan–Meier analysis, there was no significant difference in the incidence of AKI among the three NUC groups in the overall cohort (*p* = 0.104, Figure 2A), or in patients with CKD stage 1 (*p* = 0.587, Figure 2B), CKD stage 2 (*p* = 0.266, Figure 2C) and CKD stage 3–5 (*p* = 0.776, Figure 2D). By multivariate analysis, cisplatin use (hazard ratio (HR) = 1.437, p = 0.015), baseline creatinine (HR = 1.384, *p* < 0.001), albumin (HR = 0.544, *p* < 0.001) and total bilirubin levels (HR = 1.449, *p* = 0.049) were independent predictors of AKI (Table 3). In subgroup patients with cisplatin use, the incidence of AKI was comparable among patients treated with ETV, TDF and TAF (*p* = 0.898, Figure 2E), whereas in subgroup patients with serum albumin <3.7 g/dL, the TAF group had a significantly lower incidence of AKI than the ETV group (13.7% vs. 32.8%, *p* = 0.004, Figure 2F).

### 2.4. Dynamic Change in eGFR over One Year

**Figure 3:**
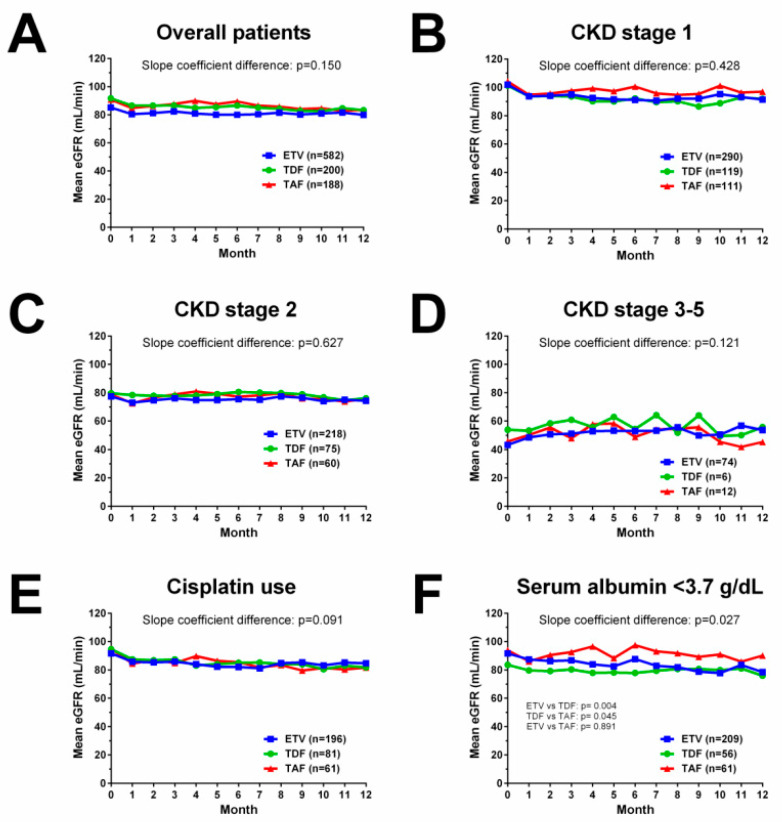
eGFR changes over 1 year during antiviral prophylaxis. (**A**) eGFR changes in overall patients. (**B**) eGFR changes in subgroup patients with CKD stage 1. (**C**) eGFR changes in subgroup patients with CKD stage 2. (**D**) eGFR changes in subgroup patients with CKD stage 3–5. (**E**) eGFR changes in subgroup patients with cisplatin use. (**F**) eGFR changes in subgroup patients with serum albumin <3.7 g/dL.

**Figure 4:**
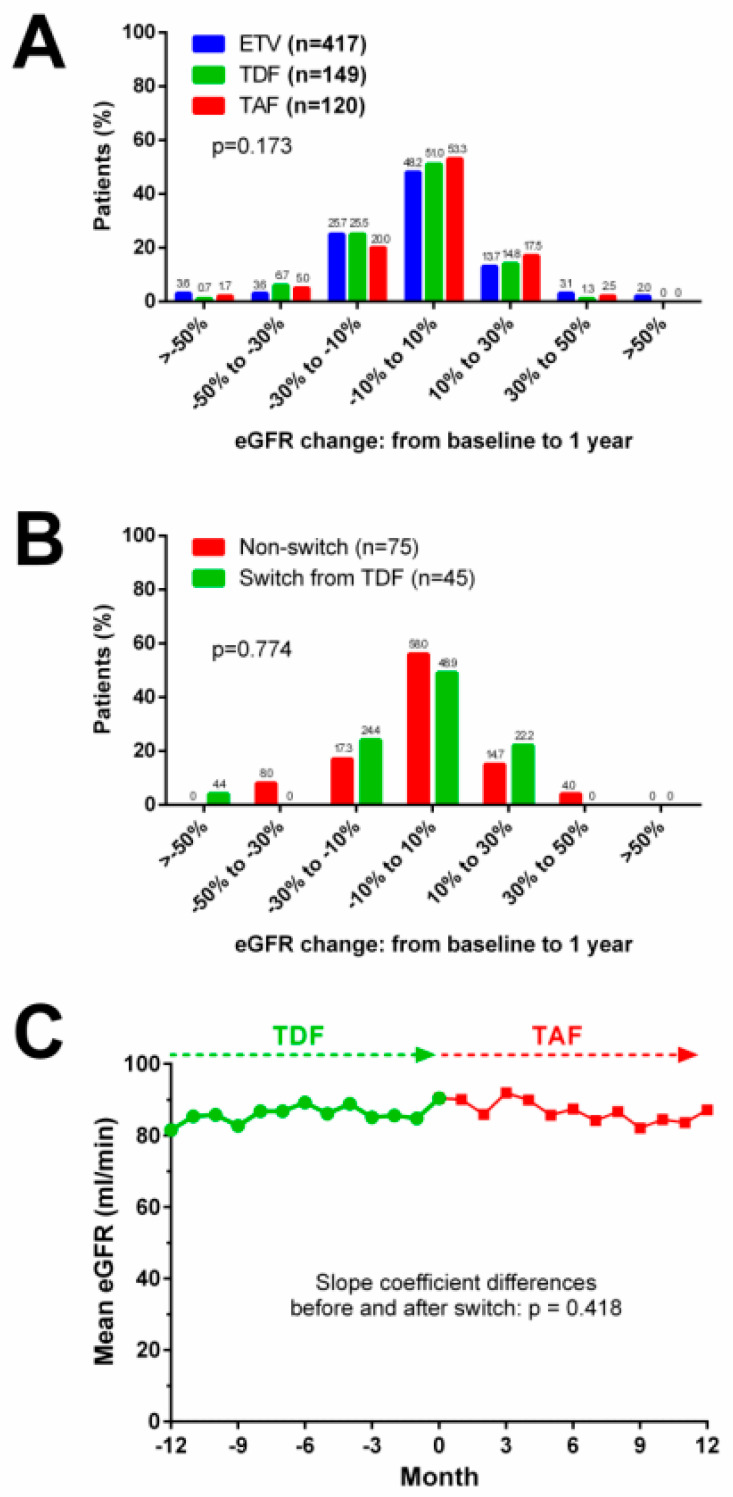
Patient distribution for eGFR changes from baseline to 1-year follow-up. (**A**) Distribution for eGFR changes in overall patients. (**B**) Distribution for eGFR changes in TAF-treated patients with and without switching from TDF. (**C**) eGFR changes in TAF-treated patients before and after switching from TDF.

The mean eGFR change over time during antiviral prophylaxis was generally stable and comparable among the ETV, TDF and TAF groups in the overall cohort (*p* = 0.150, Figure 3A), and in subgroup patients with CKD stage 1 (*p* = 0.428, Figure 3B), CKD stage 2 (*p* = 0.627, Figure 3C) and CKD stage 3–5 (*p* = 0.121, Figure 3D). The mean eGFR change was comparable among the three groups in patients with cisplatin use (Figure 3E), whereas in patients with albumin <3.7 g/dL, the TAF group had a less prominent decline in eGFR over 1 year than the ETV group (Figure 3F). The majority of patients had an eGFR change of less than 30% from baseline to 1 year, and the distributions of eGFR change were comparable among the ETV, TDF and TAF groups (*p* = 0.173, Figure 4A).

### 2.5. Renal Events in TAF-Treated Patients with and without Switching from TDF

The median duration of TDF use before switching was 11 months (interquartile range (IQR) 6.2 to 24.7 months), while the median duration of TAF use after switching was 11.3 months (IQR 4.9 to 14.6 months). In 120 TAF-treated patients with follow-up for more than 1 year, 45 of them were initially treated with TDF. The incidences of AKI, eGFR decrease, CKD stage migration and hypophosphatemia were not significantly different between patients with and without switching from TAF (Table 4). The distributions of eGFR change were comparable between patients with and without switching (Figure 4B). In patients who had switched from TDF to TAF, the mean eGFR changes were stable throughout 1 year before and after switching (*p* = 0.418, Figure 4C).

## 3. Discussion

ETV, TDF and TAF are the first-line treatment options for antiviral prophylaxis for HBV-infective patients undergoing chemotherapy [6,7,8], but currently there are no data reporting the efficacy and safety of TAF in this setting. This is the first study to compare the efficacy and renal safety of TAF with ETV and TDF during chemotherapy. Our results showed that TAF therapy had comparable antiviral efficacy with ETV and TDF, and had good renal safety, especially in patients with a high risk of AKI during chemotherapy. We also showed that switching from TDF to TAF was safe, with well-maintained efficacy in the chemotherapy setting.

We observed that patients in the TDF group were significantly younger and had higher baseline eGFR, and there were fewer patients with an advanced CKD stage. In the past, due to the potential nephrotoxicity of TDF [10], patients with older age and renal dysfunction rarely received TDF treatment during chemotherapy. Recently, several studies have reported the improved renal safety profile of TAF due to higher plasma stability, and no dosage adjustment is required in patients with eGFR >15 mL/min, or in patients with eGFR < 15 mL/min who are receiving hemodialysis [9]. Therefore, TAF is now increasingly used instead of TDF during chemotherapy, including in patients with advanced kidney diseases.

The comparable rate of TDF and TAF in achieving undetectable HBV DNA has been shown by phase III trials [11,12] and real-world studies [16]. Although few patients developed HBV reactivation, poor compliance with drug interruptions was found in these cases, while none of them showed the emergence of drug resistance. Recent studies showed that TAF was effective and could be substituted for TDF in patients with multidrug-resistant HBV [17]. Therefore, TAF could be a preferred option for patients with concerns of drug resistance, especially in patients with prior NUC exposure [7,8].

AKI during chemotherapy is associated with increased morbidity and mortality and may lead to the interruption of chemotherapy [14]. Fluctuations in renal function were frequently encountered during chemotherapy, and a significant proportion of patients may experience CKD stage migration and AKI during chemotherapy, especially in patients with more advanced kidney diseases. We did not find significant differences in the incidence of renal events among the ETV, TDF and TAF groups, except that the incidence of eGFR below 50 mL/min was higher in the ETV group, which might be related to the older age and lower baseline eGFR in the ETV group. eGFR falling below 50 mL/min is a clinically relevant event because dose adjustments of ETV and TDF are warranted in patients with eGFR below this level. Notably, no dosage adjustment of TAF is needed for patients with CKD stage migration, which would be an advantage of TAF since frequent fluctuations in eGFR might be encountered during chemotherapy.

Cisplatin use, baseline creatinine, total bilirubin and albumin levels were identified as independent predictors of AKI by multivariate analysis, whereas NUC type was not associated with AKI. Cisplatin is a widely used cancer chemotherapeutic agent with well-known nephrotoxicity [18]. The higher baseline total bilirubin or creatinine levels indicate a more advanced liver and kidney dysfunction, which is known to be a risk factor of AKI in CHB patients [19]. Hypoalbuminemia, which is common in cancer patients with cachexia, may lead to increased free circulating drug, thus increasing the risk of chemotherapy-related nephrotoxicity and AKI [15,18,20]. Since the free plasma concentration of NUCs might also increase in the case of lower plasma protein binding, the lower plasma concentration of TAF might be associated with better renal safety in patients with hypoalbuminemia [21]. Although there was a potential risk of nephrotoxicity of TDF, several studies showed no significant difference in the incidence of renal dysfunction between TDF and ETV treatment for CHB patients [15,22,23,24,25]. Since the risk of AKI is much higher in cancer patients receiving chemotherapy, the potential effect of NUCs on renal function might be less prominent in these patients as compared to the general CHB population.

Although a certain proportion of patients may experience fluctuations in renal function during chemotherapy, our previous study showed that most of the renal events were transient and reversible [15]. In this study, the mean eGFR change throughout 1 year was generally stable and was not significantly different among the ETV, TDF and TAF groups. In subgroup patients with cisplatin use, the risk of AKI and eGFR dynamics was comparable among ETV, TDF and TAF groups, whereas in subgroup patients with hypoalbuminemia, the TAF group had a relatively lower risk of AKI and eGFR decline, suggesting that TAF use was relatively safe in patients with predisposing factors of AKI.

Clinical trials and real-world studies have shown that TDF could be safely switched to TAF in CHB patients for improved safety without a loss of efficacy [13,26]. Consistent with previous reports, all patients who switched from TDF to TAF in this study had a well-maintained virological response after switching. We did not find a significant increase in eGFR after the switch, as reported in the previous randomized controlled trial [13]. A recent real-world study also reported no significant change in mean eGFR after switching from TDF to TAF [16]. Since the eGFR increase observed in the previous trial was only by 0.94 mL/min [13], this effect may not be evident in our cohort since many patients had significant fluctuations in eGFR during chemotherapy.

This study has some limitations. First, this is a retrospective study from a single medical center. However, liver and renal functions were routinely measured before each session of chemotherapy. Therefore, close monitoring of renal dysfunction during chemotherapy could be achieved. Second, since cancer patients with more advanced kidney disease were less likely to receive aggressive chemotherapy, the case number of patients with CKD stage 3–5 was relatively small. The renal safety of TAF for patients with an advanced CKD stage warrants further study. Third, the follow-up period in the TAF group was relatively short. The long-term efficacy and renal safety of TAF also need future study. Fourth, there were significant differences in the baseline characteristics of the three groups. The ETV group had a significantly longer chemotherapy duration and poorer renal function, leading to a higher rate of AKI and eGFR below 50 mL/min in the ETV group. Nevertheless, the multivariate analysis showed that the type of antiviral drug was not a significant factor associated with AKI.

## 4. Materials and Methods

### 4.1. Patients

From 1 April 2016 to 31 March 2020, a consecutive 1209 patients receiving NUC prophylaxis during chemotherapy in Taipei Veterans General Hospital were retrospectively screened (Figure 1). The inclusion criteria were: (1) age ≥ 20 years; (2) seropositive for HBsAg at the entry of this study; (3) undergoing systemic chemotherapy for cancer; and (4) using ETV (Baraclude, Bristol-Myers Squibb, Princeton, NJ), TDF (Viread, Gilead Sciences, Foster City, CA, USA) or TAF (Vemlidy, Gilead Sciences, Foster City, CA, USA) for prophylaxis. The exclusion criteria were: (1) NUC switching from TDF to ETV (n = 11); (2) NUC switching from ETV to TAF (n = 7); (3) presence of cirrhosis (n = 15); (4) died or lost to follow-up within 3 months after starting chemotherapy (n = 206).

Under the regulations of National Health Insurance Administration, Ministry of Health and Welfare, Taiwan, as well as the recommendations of the American Association for the Study of Liver Diseases (AASLD) treatment guidelines, prophylactic NUC antiviral prophylaxis was prescribed from 1 week prior to starting chemotherapy until 6 months after the cessation of chemotherapy, and the HBV DNA level was monitored at 6-month intervals during NUC therapy [8]. In patients with renal dysfunction, the dose of ETV or TDF was adjusted based on eGFR [27].

This study was approved by the Institutional Review Board, Taipei Veterans General Hospital (IRB number: 2021-03-008AC), which complied with standards of the Declaration of Helsinki and current ethical guidelines. Due to the retrospective nature of the study, the Institutional Review Board waived the need for written informed consent.

### 4.2. Biochemistry and Virological Tests

The following parameters were collected: age, sex, body mass index (BMI), diabetes mellitus (DM), hypertension, cancer types, duration and regimen of chemotherapy, serum HBV DNA, HBsAg, HBeAg, BUN, creatinine, albumin, total bilirubin, alanine aminotransferase (ALT) and aspartate aminotransferase (AST) levels. Serum biochemistry tests were performed using a systemic multi-autoanalyzer (Technicon SMAC, Technicon Instruments Corp., Tarrytown, NY, USA). HBV DNA levels were determined by Roche Cobas Taqman HBV DNA assay (detection limit of 20 IU/mL, Roche Diagnostics, Switzerland). Quantitative HBsAg level was measured by the Elecsys HBsAg II assay (Roche Diagnostics, Mannheim, Germany) or Abbott Architect HBsAg assay (Abbott Diagnostics, Abbott Park, IL, USA) with a detection limit of 0.05 IU/mL.

### 4.3. Outcomes

Renal events were assessed by serum creatinine increases and changes in eGFR, which were calculated using the Chronic Kidney Disease Epidemiology Collaboration (CKD-EPI) equation [28]. AKI was defined according to the Kidney Disease: Improving Global Outcomes (KDIGO) clinical practice guidelines [29]. Hypophosphatemia was defined as serum phosphorus less than 2 mg/dL. Time to renal events was calculated from the time of starting NUC therapy to the time of developing renal events for survival analysis. Reactivation of HBV was defined according to the AASLD criteria [8]. Virological response was defined as achieving undetectable HBV DNA after NUC therapy.

### 4.4. Statistical Analysis

Values were expressed as mean ± standard deviation or as median (IQR) when appropriate. Continuous variables were compared by Mann–Whitney U test or Kruskal–Wallis test. Categorical variables were compared by Pearson chi-square analysis or the Fisher exact test. The time to renal events was estimated by the Kaplan–Meier method. The survival curves between patient groups were compared by log-rank test. The Cox proportional hazards model was used to analyze prognostic factors of renal events. Variables with statistical significance (*p* < 0.05) or close to significance (*p* < 0.1) in univariate analysis were included in multivariate analysis using the forward stepwise Cox proportional-hazards model. A generalized linear mixed-effects model was used to evaluate the slope coefficient differences [30]. A two-tailed *p* < 0.05 was considered statistically significant. All statistical analyses were performed using IBM SPSS Statistics V22 (IBM, Armonk, NY, USA).

## 5. Conclusions

In conclusion, in HBV-infected cancer patients receiving chemotherapy, TAF had comparable antiviral efficacy to ETV and TDF. TAF also had relatively good renal safety and the advantage of not requiring dosage adjustment in the case of fluctuations in renal function, which could be frequently encountered during chemotherapy. Switching from TDF to TAF during chemotherapy is also safe, without a loss of efficacy.

## Figures and Tables

**Table 1:** Baseline characteristics of the 970 HBV-infected cancer patients receiving chemotherapy.

	ETV (n = 582, 60%)	TDF (n = 200, 20.6%)	TAF (n = 188, 19.4%)	*p*
Age (years)	59.4 ± 12.2	56.9 ± 11.7	58.6 ± 11.1	0.024
Sex (male), n (%)	267 (45.9)	99 (49.5)	79 (42.0)	0.385
Body mass index (kg/m^2^)	23.6 ± 3.8	23.8 ± 4.5	23.5 ± 4.1	0.623
Diabetes, n (%)	79 (13.6)	27 (13.5)	20 (10.6)	0.565
Hypertension, n (%)	154 (26.5)	57 (28.5)	45 (23.9)	0.594
Cancer types, n (%)				<0.001
Gastrointestinal cancers	114 (19.6)	65 (32.5)	59 (31.4)	
Hematological cancers	106 (18.2)	17 (8.5)	26 (13.6)	
Lung cancer	93 (16)	30 (15)	25 (13.3)	
Head and neck cancers	66 (11.3)	29 (14.5)	13 (6.9)	
Breast cancer	64 (11)	33 (16.5)	24 (12.8)	
Hepatobiliary cancer	39 (6.7)	7 (3.5)	4 (2.1)	
Gynecological cancer	43 (7.4)	9 (4.5)	15 (8)	
Others	57 (9.8)	10 (5)	22 (11.7)	
Chemotherapy duration (months)	7.7 (4.2–18.4)	6.0 (4.0–11.6)	6.4 (3.7–11.7)	0.001
Chemotherapy regimen, n (%)				
Rituximab-containing	66 (11.3)	9 (4.5)	21 (11.2)	0.016
Platinum-containing	298 (51.2)	108 (54)	102 (54.3)	0.671
Cisplatin-containing	196 (33.7)	81 (40.5)	61 (32.4)	0.162
Concurrent radiotherapy	157 (27)	60 (30)	45 (23.9)	0.405
NUC therapy duration (months)	12.4 (8–21.6)	10.6 (8.2–17)	10.2 (7.7–13.2)	<0.001
Ongoing NUC therapy, n (%)	162 (27.8)	7 (3.5)	122 (64.9)	<0.001
Follow-up period (months)	18.4 (10.2–35.1)	20 (10.7–40.5)	11.9 ± 3.5	<0.001
Death during follow-up period	169 (29)	116 (42)	15 (8)	<0.001
HBV DNA (Log IU/mL) *	2.99 ± 1.68	2.78 ± 1.57	2.93 ± 1.85	0.071
Undetectable HBV DNA, n (%) *	134 (23.9)	49 (25.7)	53 (30.5)	0.220
HBV DNA < 2000 IU/mL, n (%) *	350 (62.4)	128 (67)	112 (64.4)	0.507
HBsAg (Log IU/mL) *	1.95 ± 1.43	1.71 ± 1.52	2.06 ± 1.38	0.173
HBV status, n (%)				0.080
HBeAg-positive carrier	22 (3.8)	5 (2.5)	10 (5.3)	
HBeAg-positive chronic hepatitis	10 (1.7)	0 (0)	1 (0.5)	
HBeAg-negative carrier	510 (87.6)	189 (94.5)	165 (87.8)	
HBeAg-negative chronic hepatitis	40 (6.9)	6 (3)	12 (6.4)	
BUN (mg/dL)	15.9 ± 9.2	13.6 ± 4.4	14.2 ± 5.8	0.015
Creatinine (mg/dL)	0.93 ± 0.54	0.81 ± 0.16	0.81 ± 0.25	<0.001
eGFR (mL/min)	80.5 ± 23.4	86.6 ± 18.9	84.9 ± 23.1	0.001
Chronic kidney disease (CKD) stage 1/2/3/4/5, n (%)	290/218/63/7/4 (49.8/37.5/10.8/1.2/0.7)	119/75/6/0/0 (59.5/37.5/3/0/0)	111/60/15/2/0 (59/31.9/8/1.1/0)	0.008
Albumin (g/dL)	3.78 ± 0.54	3.86 ± 0.46	3.80 ± 0.57	0.166
Total bilirubin (mg/dL)	0.59 ± 0.35	0.61 ± 0.34	0.57 ± 0.36	0.308
ALT (U/L)	40.5 ± 86.0	29.1 ± 32.8	32.9 ± 41.1	0.575
AST (U/L)	36.1 ± 52.1	29.6 ± 27.1	30.1 ± 26.1	0.093

ETV, entecavir; TDF, tenofovir disoproxil fumarate; TAF, tenofovir alafenamide. * 926 (95.5%) cases had available baseline HBV DNA level.

**Table 2:** Antiviral efficacy and incidence of renal events at 1 year after starting NUC therapy in 686 patients with follow-up of more than 1 year.

Events, n (%)	ETV (n = 417, 60.8%)	TDF (n = 149, 21.7%)	TAF (n = 120, 17.5%)	*p*
**Antiviral efficacy**				
Virological response *	250 (94.7)	89 (94.7)	74 (96.1)	0.877
HBV reactivation	2 (0.5)	1 (0.7)	0 (0)	0.694
**Renal events—all CKD stages**				
Acute kidney injury	61 (14.6)	17 (11.4)	13 (10.8)	0.420
eGFR decrease > 30%	121 (29)	40 (26.8)	24 (20)	0.146
eGFR < 50 mL/min	101 (24.2)	13 (8.7)	16 (13.3)	<0.001
Dose reduction	14 (13.9)	5 (38.5)	-	0.041
≥1 stage worsening in CKD stage at 1 year	56 (13.4)	21 (14.1)	12 (10)	0.554
≥1 stage improvement in CKD stage at 1 year	52 (12.5)	15 (10.1)	14 (11.7)	0.737
Serum phosphorus < 2 mg/dL	64 (15.3)	17 (11.4)	14 (11.7)	0.367
**Renal events—CKD stage 1**				
Case number	213	85	62	
Acute kidney injury	25 (11.7)	9 (10.6)	4 (6.5)	0.491
eGFR decrease > 30%	58 (27.2)	25 (29.4)	8 (12.9)	0.044
eGFR < 50 mL/min	18 (8.5)	2 (2.4)	2 (3.2)	0.081
Dose reduction	1 (5.6)	1 (50)	-	0.195
≥1 stage worsening in CKD stage at 1 year	54 (25.4)	21 (24.7)	9 (14.5)	0.195
Serum phosphorus < 2 mg/dL	34 (16)	7 (8.2)	7 (11.3)	0.182
**Renal events—CKD stage 2**				
Case number	157	60	49	
Acute kidney injury	25 (15.9)	7 (11.7)	6 (12.2)	0.655
eGFR decrease > 30%	54 (34.4)	13 (21.7)	13 (26.5)	0.157
eGFR < 50 mL/min	45 (28.7)	8 (13.3)	8 (16.3)	0.027
Dose reduction	2 (4.4)	2 (25)	-	0.104
≥1 stage worsening in CKD stage at 1 year	1 (0.6)	0 (0)	2 (4.1)	0.088
≥1 stage improvement in CKD stage at 1 year	34 (21.7)	14 (23.3)	11 (22.4)	0.964
Serum phosphorus < 2 mg/dL	23 (14.6)	9 (15)	6 (12.2)	0.901
**Renal events—CKD stage 3–5**				
Case number	47	4	9	
Acute kidney injury	11 (23.4)	1 (25)	3 (33.3)	0.820
eGFR decrease > 30%	9 (19.1)	2 (50)	3 (33.3)	0.279
eGFR < 50 mL/min	38 (80.9)	3 (75)	6 (66.6)	0.630
Dose reduction	11 (28.9)	2 (66.7)	-	0.232
≥1 stage worsening in CKD stage at 1 year	1 (2.1)	0 (0)	1 (11.1)	0.361
≥1 stage improvement in CKD stage at 1 year	18 (38.3)	1 (25)	3 (33.3)	0.847
Serum phosphorus < 2 mg/dL	7 (14.9)	1 (25)	9 (100)	0.810

ETV, entecavir; TDF, tenofovir disoproxil fumarate; TAF, tenofovir alafenamide. * 435 (63.4%) patients had available follow-up HBV DNA data.

**Table 3:** Univariate and multivariate analyses of factors associated with acute kidney injury during antiviral prophylaxis.

	Univariate			Multivariate		
	HR	95% CI	*p*	HR	95% CI	*p*
Age (years)	1.014	1.003–1.025	0.015			NS
Sex (male)	0.688	0.520–0.909	0.009			NS
Diabetes (yes vs. no)	1.428	0.993–2.053	0.055			NS
Hypertension (yes vs. no)	1.366	1.019–1.832	0.037			NS
Hematologic cancer (yes vs. no)	1.245	0.875–1.769	0.223			
Platinum-based chemotherapy (yes vs. no)	1.095	0.83–1.438	0.516			
Cisplatin-based chemotherapy (yes vs. no)	1.477	1.122–1.944	0.005	1.437	1.072–1.925	0.015
Radiotherapy (yes vs. no)	1.128	0.836–1.523	0.430			
HBeAg-positive (yes vs. no)	0.973	0.515–1.837	0.933			
HBV DNA (Log IU/mL)	1.014	0.933–1.101	0.751			
HBsAg (Log IU/mL)	0.974	0.881–1.077	0.613			
Body mass index (kg/m^2^)	1.013	0.980–1.048	0.438			
BUN (mg/dL)	1.026	1.011–1.040	<0.001			NS
Creatinine (mg/dL)	1.501	1.300–1.733	<0.001	1.384	1.164–1.646	<0.001
eGFR	0.990	0.983–0.996	0.001			NS
ALT (U/L)	0.999	0.997–1.002	0.531			
AST (U/L)	1.000	0.998–1.003	0.726			
Albumin (g/dL)	0.520	0.407–0.664	<0.001	0.544	0.426–0.696	<0.001
Total bilirubin (mg/dL)	1.442	1.014–2.052	0.042	1.449	1.002–2.096	0.049
WBC count	1.000	1.000–1.000	0.924			
Hemoglobin	0.885	0.827–0.946	<0.001			NS
Platelet count	1.000	1.000–1.000	0.692			
NUCs therapy						
ETV	1		0.026			NS
TDF	0.848	0.603–1.193	0.345			
TAF	0.549	0.353–0.855	0.008			

HR, hazard ratio; CI, confidence interval; NS, not significant; NUCs, nucleos(t)ide analogues; ETV, entecavir; TDF, tenofovir disoproxil fumarate; TAF, tenofovir alafenamide; BUN, blood urea nitrogen.

**Table 4:** Incidence of renal events at 1 year in 120 TAF-treated patients with follow-up of more than 1 year, with and without switching from TDF.

Renal Events, n (%)	No Switching (n = 75)	Switching from TDF (n = 45)	*p*
Acute kidney injury	8 (10.7)	5 (11.1)	1.000
eGFR decrease > 30%	18 (24)	6 (13.3)	0.239
eGFR < 50 mL/min	11 (14.7)	5 (11.1)	0.782
≥1 stage worsening in CKD stage at 1 year	7 (9.3)	5 (11.1)	0.762
≥1 stage improvement in CKD stage at 1 year	10 (13.3)	4 (8.9)	0.660
Serum phosphorus < 2 mg/dL	7 (9.3)	7 (15.6)	0.463

## Data Availability

The data that support the findings of this study are available from the corresponding author upon reasonable request.

## Supplementary Materials

The following supporting information can be downloaded at: https://www.mdpi.com/article/10.3390/ijms231911335/s1.

## Abbreviations

TAF: tenofovir alafenamide; TDF, tenofovir disoproxil fumarate; ETV, entecavir; HBsAg, hepatitis B surface antigen; eGFR, estimated glomerular filtration rate; CKD, chronic kidney disease; CKD-EPI, Chronic Kidney Disease Epidemiology Collaboration; HR, hazard ratio; HBV, hepatitis B virus; NUCs, nucleos(t)ide analogues; CHB, chronic hepatitis B; DM, diabetes mellitus; APASL, Asian Pacific Association for the Study of the Liver; ALT, alanine aminotransferase; AST, aspartate aminotransferase; AKI, acute kidney injury; AASLD, American Association for the Study of Liver Diseases; KDIGO, Kidney Disease: Improving Global Outcomes.
