# Supplementary material for: Efficacy and Renal Safety of Prophylactic Tenofovir Alafenamide for HBV-Infected Cancer Patients Undergoing Chemotherapy
Source: Int J Mol Sci. 2022 Sep 26;23(19):11335. doi: 10.3390/ijms231911335 (PMC9569539; doi:10.3390/ijms231911335)
Supplement: Supplementary file 1 [file ijms-23-11335-s001.zip › TAF chemo Tables S1 20220905.pdf]

Table S1. Baseline characteristics of the 686 HBV-infected cancer patients with follow-up more than 1 year

	ETV (n=417, 60.8%)	TDF (n=149, 21.7%)	TAF (n=120, 17.5%)	***p***
Age (years)	58.4 ± 11.8	56.0 ± 11.8	58.6 ± 11.1	0.054
Sex (male), n (%)	233 (55.9)	71 (47.7)	71 (59.2)	0.123
Body mass index (kg/m^2)	23.8 ± 3.8	23.8 ± 4.6	23.6 ± 3.9	0.429
Diabetes, n (%)	57 (13.7)	22 (14.8)	12 (10)	0.481
Hypertension, n (%)	101 (24.2)	42 (28.2)	27 (22.5)	0.513
Cancer types, n (%)				0.001
Gastrointestinal cancers	81 (19.4)	46 (30.9)	41 (34.2)	
Hematological cancers	75 (18)	12 (8.1)	15 (12.5)	
Lung cancer	65 (15.6)	22 (14.8)	16 (13.3)	
Head and neck cancers	48 (11.5)	22 (14.8)	9 (7.5)	
Breast cancer	54 (12.9)	30 (20.1)	16 (13.3)	
Hepatobiliary cancer	17 (4.1)	4 (2.7)	1 (0.8)	
Gynecological cancer	31 (7.4)	7 (4.7)	8 (6.7)	
Others	46 (11)	6 (4)	14 (11.7)	
Chemotherapy duration (months)	11.5 (4.9-22.6)	6.1 (4.3-14.7)	8.1 (5.0-14.0)	0.001
Chemotherapy regimen, n (%)				
Rituximab-containing	45 (10.8)	7 (4.7)	10 (8.3)	0.080
Platinum-containing	215 (51.6)	87 (58.4)	69 (57.5)	0.253
Cisplatin-containing	136 (32.6)	62 (41.6)	39 (32.5)	0.122
Concurrent radiotherapy	113 (27.1)	45 (30.2)	33 (27.5)	0.765
NUC therapy duration (months)	16.4 (11.6-25.6)	12.5 (9.3-20.2)	12.8 (9.4-14.2)	<0.001

Ongoing NUCs therapy, n (%)	122 (29.3)	6 (4)	74 (61.7)	<0.001
Follow-up period (months)	24.9 (16.9-41.4)	27.8 (18.4-44.7)	14.0 (13.1-15.4)	<0.001
Death during follow-up period	86 (20.6)	41 (27.5)	1 (0.8)	<0.001
HBV DNA (Log IU/mL)*	3.03 ± 1.70	2.68 ± 1.38	2.87 ± 1.79	0.034
Undetectable HBV DNA, n (%)*	95 (23.6)	36 (25.2)	35 (32.4)	0.173
HBV DNA <2000 IU/mL, n (%)*	244 (60.5)	99 (69.2)	71 (65.7)	0.153
HBsAg (Log IU/mL)*	1.95 ± 1.42	1.73 ± 1.53	2.09 ± 1.42	0.237
HBV status, n (%)				0.078
HBeAg-positive carrier	13 (3.1)	4 (2.7)	7 (5.8)	
HBeAg-positive chronic hepatitis	9 (2.2)	0 (0)	1 (0.8)	
HBeAg-negative carrier	368 (88.2)	142 (95.3)	107 (89.2)	
HBeAg-negative chronic hepatitis	27 (6.5)	3 (2)	5 (4.2)	
BUN (mg/dL)	15.6 ± 8.8	13.7 ± 4.2	14.0 ± 5.5	0.150
Creatinine (mg/dL)	0.93 ± 0.56	0.81 ± 0.16	0.83 ± 0.24	0.018
eGFR (mL/min)	86.2 ± 21.2	91.7 ± 14.6	87.7 ± 17.9	0.072
Chronic kidney disease (CKD) stage 1/2/3/4/5, n (%)	213/157/41/3/3 (51.1/37.6/9.8/0.7/0.7)	85/60/4/0/0 (57/40.3/2.7/0/0)	62/49/7/2/0 (51.7/40.8/5.8/1.7/0)	0.094
Albumin (g/dL)	3.82 ± 0.54	3.93 ± 0.43	3.86 ± 0.52	0.146
Total bilirubin (mg/dL)	0.59 ± 0.35	0.58 ± 0.31	0.59 ± 0.33	0.954
ALT (U/L)	42 ± 97	25 ± 18	31 ± 34	0.256
AST (U/L)	35 ± 56	26 ± 17	28 ± 23	0.052

ETV, entecavir; TDF, tenofovir disoproxil fumarate; TAF, tenofovir alafenamide.

*654 (95.3%) cases had available baseline HBV DNA level.
